# Convolution Operations on Coding Metasurface to Reach Flexible and Continuous Controls of Terahertz Beams

**DOI:** 10.1002/advs.201600156

**Published:** 2016-07-07

**Authors:** Shuo Liu, Tie Jun Cui, Lei Zhang, Quan Xu, Qiu Wang, Xiang Wan, Jian Qiang Gu, Wen Xuan Tang, Mei Qing Qi, Jia Guang Han, Wei Li Zhang, Xiao Yang Zhou, Qiang Cheng

**Affiliations:** ^1^ State Key Laboratory of Millimeter WavesSoutheast UniversityNanjing210096China; ^2^ Synergetic Innovation Center of Wireless Communication TechnologySoutheast UniversityNanjing210096China; ^3^ Cooperative Innovation Centre of Terahertz ScienceNo.4, Section 2, North Jianshe RoadChengdu610054China; ^4^ Center for Terahertz Waves and College of Precision Instrument and Optoelectronics EngineeringTianjin UniversityTianjin300072China; ^5^ Jiangsu Xuantu Technology Co. Ltd12 Mozhou East RoadNanjing211111China

**Keywords:** coding metasurface, convolution theorem, Fourier transform, scattering‐pattern shift, terahertz

## Abstract

The concept of coding metasurface makes a link between physically metamaterial particles and digital codes, and hence it is possible to perform digital signal processing on the coding metasurface to realize unusual physical phenomena. Here, this study presents to perform Fourier operations on coding metasurfaces and proposes a principle called as scattering‐pattern shift using the convolution theorem, which allows steering of the scattering pattern to an arbitrarily predesigned direction. Owing to the constant reflection amplitude of coding particles, the required coding pattern can be simply achieved by the modulus of two coding matrices. This study demonstrates that the scattering patterns that are directly calculated from the coding pattern using the Fourier transform have excellent agreements to the numerical simulations based on realistic coding structures, providing an efficient method in optimizing coding patterns to achieve predesigned scattering beams. The most important advantage of this approach over the previous schemes in producing anomalous single‐beam scattering is its flexible and continuous controls to arbitrary directions. This work opens a new route to study metamaterial from a fully digital perspective, predicting the possibility of combining conventional theorems in digital signal processing with the coding metasurface to realize more powerful manipulations of electromagnetic waves.

## 1 Introduction

As 2D artificially engineered structures,^1^, ^2^, ^3^ metasurfaces have attracted great attention in physics and engineering communities owing to a number of unique properties.^4^, ^5^, ^6^, ^7^, ^8^, ^9^, ^10^, ^11^, ^12^ A metasurface is formed by distributing subwavelength resonant particles with different geometries and materials on a 2D surface, and therefore is able to manipulate both amplitudes and phases of electromagnetic (EM) waves, enabling many extraordinary functionalities such as the polarization conversion,,^13^, ^14^, ^15^, ^16^, ^17^ perfect absorption,^18^, ^19^, ^20^ and amplitude and phase modulations.^21^, ^22^ The generalized Snell's law proposed in 2001^23^ has sped up the development of metasurfaces in the past a few years, enabling a lot of interesting devices to manipulate microwaves,^24^, ^25^, ^26^ terahertz waves,^27^, ^28^ and visible lights.^29^, ^30^, ^31^

Recently, the concepts of coding, digital, and programmable metamaterials/metasurfaces have been proposed.^32^, ^33^ In coding metamaterials, the EM properties are no longer described by the effective medium parameters with continuous values.^33^ It has been shown that binary codes “0” and “1” can be used to characterize the reflection phases 0° and 180° of the coding particles, which are distributed on a 2D surface with certain coding schemes to realize various manipulations to EM waves, such as the anomalous beam reflections and random diffusions.^34^, ^35^, ^36^ This idea was later extended to the anisotropic case, producing anisotropic coding metamaterials which enable the independent controls of THz waves under orthogonal polarizations.^37^ The concept of coding metasurface makes a link between physically metamaterial particles and digital codes, and hence it is possible to perform digital signal processing on the coding metasurface to realize a lot of unusual physical phenomena.

In the previous work on coding metamaterials,^33^ several examples have been given to show the powerful manipulations to EM waves using a few simple coding sequences. It has been shown that a 2‐bit coding metasurface is able to generate a single‐beam radiation under the normal illumination, whose anomalously reflected angle is determined by the periodicity of a gradient coding sequence “0 1 2 3 0 1 2 3 …,” i.e., the minimum physical length of the gradient phase distribution. It is generally considered that a continuous phase profile is required to produce a single beam with arbitrary radiation angle. Using the coding metasurface, however, only a limited number of anomalously deflected angles (especially for large deflected angles) can be achieved because the minimum gradient coding sequence is multiplied with a discrete integer but not a continuous real number. Although the reflection phase can be designed continuously from 0 to 2π, it is practically impossible to design and fabricate a coding particle with infinitely small dimension.

In this article, we present to perform Fourier operations, the simplest digital signal processing, on the coding metasurfaces, and propose a principle called as scattering‐pattern shift using the most famous convolution theorem. The proposed principle and method can make flexible and continuous manipulations to scattering beams (or patterns) of EM waves using the coding metasurfaces. For the particular design in this article, we can realize a single scattering beam and steer it to an arbitrarily pre‐designed direction with negligible distortion to the shape of coding pattern. We further show that this idea can be easily implemented with coding metasurface by calculating the modulus of the original coding sequence and a gradient coding sequence. The flexible and continuous controls of the far‐field EM waves by the proposed principle have been demonstrated theoretically and numerically with several examples, and verified experimentally by four different samples fabricated at terahertz frequencies.

## 2 Principle of Scattering‐Pattern Shift

Before introducing the principle of scattering‐pattern shift, we note that the scattering pattern can be analytically calculated by the Fourier transform once the coding pattern on a coding metasurface is given (see the Supporting Information and Figure S1 for detailed derivation)

(1) $$E\left(\theta ,\varphi \right)=jk\left(\hat{\theta }\cos\varphi -\hat{\varphi }\sin\varphi \cos\theta \right)P\left(u,v\right)$$

where E(θ,φ) represents the scattered electric field in the far‐field region at distance *r*, elevation angle *θ*, and azimuth angle *ϕ* in the spherical coordinate; *k* is the wavenumber in free space; and *P*(*u*, *v*) is the 2D Fourier transform of tangential electric field *E*(*x*, *y*) on the coding metasurface

(2) $$P\left(u,v\right)=\overset{\frac{Np}{2}}{\underset{-\frac{Np}{2}}{\;\int }}\overset{\frac{Np}{2}}{\underset{-\frac{Np}{2}}{\;\int }}E\left(x,y\right)e^{jk_{0}\left(ux+vy\right)}dxdy$$

where *u*, *v* are the angular coordinates. In the product item *Np*, *N* is the number of coding particles along the *x* and *y* directions, and *p* is the side‐length of the coding particle. The scattering pattern can be expressed by the coordinates (θ,φ) by making a coordinate transformation from (*u*, *v*) to (θ,φ)

(3) u=sinθcosφ, v=sinθsinφ

We know that the Fourier transform decomposes a function of time (a signal) into the frequencies that make it up, i.e., the frequency domain representation of the original signal. In this regard, Equation (1) connects the coding‐pattern domain (in analogy to the time domain) with the scattering‐pattern domain (in analogy to the frequency domain), and thereby mimics the Fourier transform of a signal from time domain to frequency domain. We remark that the idea of linking the coding pattern to scattering pattern through Fourier transform enables us to design the coding pattern from a new perspective, since many existing properties of Fourier transform could be potentially applied to the analyses of coding metasurfaces. One of the most interesting properties of Fourier transform is the theorem of convolution, which describes the equivalence principle between the ordinary multiplication of two signals in time domain and the convolution of their corresponding frequency spectra in the frequency domain, as is mathematically expressed as

(4) $$f\left(t\right)\cdot g\left(t\right)\overset{\mathrm{FFT}}{\Leftrightarrow }f\left(\omega \right)*g\left(\omega \right)$$

Owing to the fact that the electric field distribution on the coding metasurface and the scattering pattern in the far‐field region are a Fourier transform pair, we could therefore replace the arguments *t* and *ω* in Equation (4) with $x_{\lambda }$ and sinθ, respectively, giving the following equation

(5) $$f\left(x_{\lambda }\right)\cdot g\left(x_{\lambda }\right)\overset{\mathrm{FFT}}{\Leftrightarrow }f\left(\sin\theta \right)*g\left(\sin\theta \right)$$

in which $x_{\lambda }=x/\lambda$ is the electrical length, and *θ* is the angle with respect to the normal direction. Note that the above equation cannot be directly applied to the design of coding metasurface because the coding digits will not be preserved after the operation of convolution, i.e., the reflection amplitudes of all coding particles are not always be equal. Fortunately, Equation (4) can be simplified as a frequency‐shift function when the item g(ω) becomes a Dirac‐delta function

(6) $$f\left(t\right)\cdot e^{j\omega_{0}t}\overset{\mathrm{FFT}}{\Leftrightarrow }f\left(\omega \right)*\delta \left(\omega -\omega_{0}\right)\;\;=\;\;f\left(\omega -\omega_{0}\right)$$

in which $e^{j\omega_{0}t}$ is the time‐shift item in the time domain and the impulse function$\;\delta (\omega_{0})$ is its frequency spectrum expression in the frequency domain. Equation (6) implies that the convolution of a spectrum f(ω) with an impulse function $\delta (\omega -\omega_{0})$ will shift the spectrum function f(ω) by $\omega_{0}$ in the frequency domain without distortion. Applying the same argument substitutions to Equation (5), we have the following function defining the principle of scattering‐pattern shift

(7) $$\begin{array}{l} \;E\left(x_{\lambda }\right)\cdot e^{jx_{\lambda }\sin\theta_{0}}\overset{\mathrm{FFT}}{\Leftrightarrow }E\left(\sin\theta \right)*\delta \left(\sin\theta -\sin\theta_{0}\right)\\ \;\;\;\;\;\;\;=E\left(\sin\theta -\sin\theta_{0}\right) \end{array}$$

in which the item $e^{jx_{\lambda }\sin\theta_{0}}$ describes the electric field distribution with unity amplitude and gradient phase along a certain direction, which can be simply implemented by the coding metasurface. The principle of scattering‐pattern shift mathematically defined in Equation (7) can be understood in the same manner as how we interpret the frequency‐shift function in Equation (6). The multiplication of a coding pattern $E(x_{\lambda })$ by a gradient coding sequence $e^{jx_{\lambda }\sin\theta_{0}}$ leads to a deviation of the scattering pattern E(sinθ) away from its original direction by the quantity $\sin\theta_{0}$ in the angular coordinate. It is interesting to note that the multiplication of the phases of two coding patterns is just equivalent to the modulus of their coding digits, providing much reduced computation complexity for the optimization of scattering patterns.

**Figure 1:**
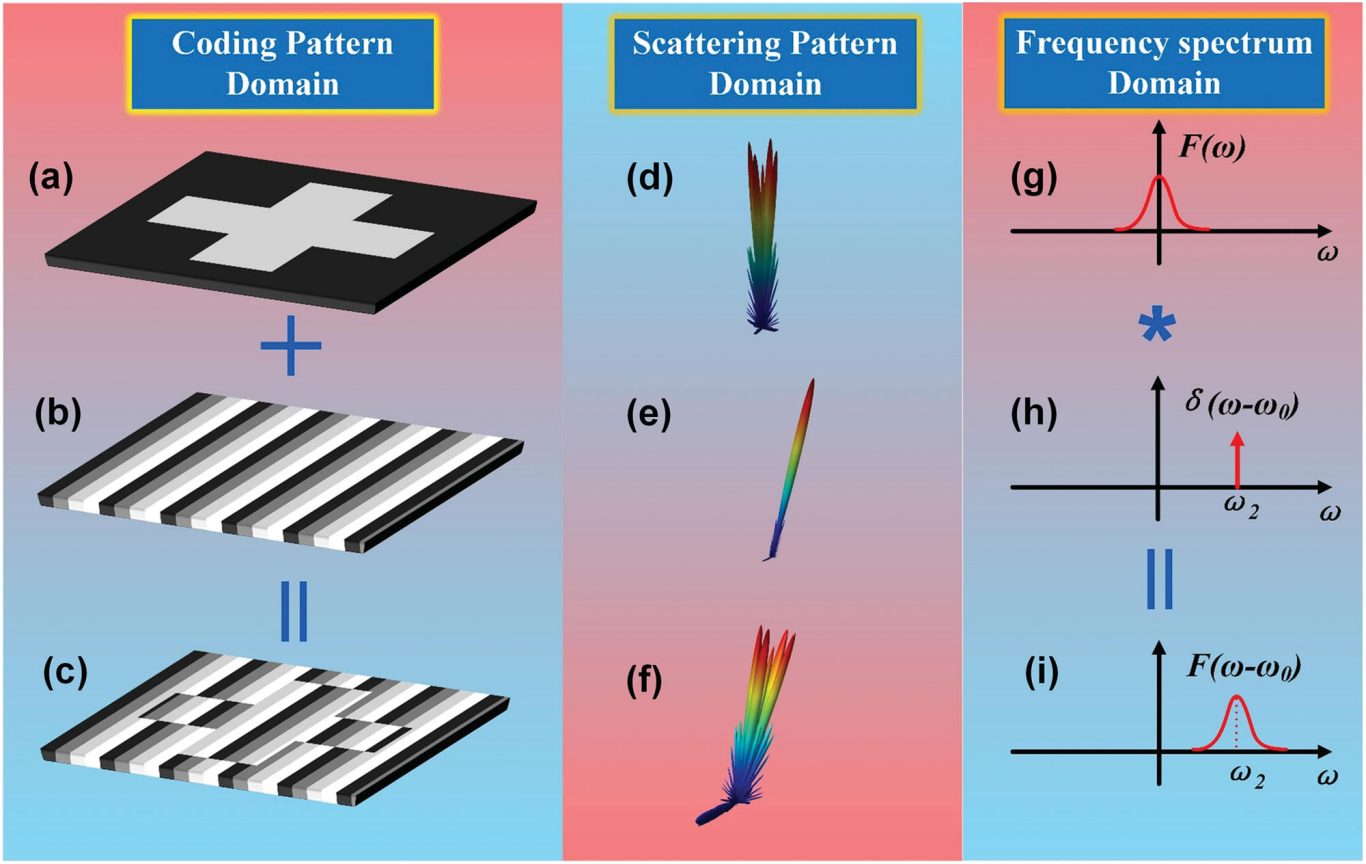
Schematic illustration to the principle of scattering‐pattern shift in analogy to the Fourier Transform. a–c) Coding patterns of a cross, gradient distribution, and their modulus, respectively. d–f) Scattering patterns calculated by FFT from the coding patterns in panels (a)–(c), respectively. g–h) The analogical frequency spectra of the coding patterns in panels (a)–(c), respectively.

We take a 2‐bit coding metasurface as a proof of principle to demonstrate how the above concept is used to control the direction of scattering pattern. **Figure** **1** shows three different coding patterns (a–c), the corresponding scattering patterns (d–f), and the analogical frequency spectra (g–i) in the left, middle, and right panels, respectively. In this illustration, the cross‐shaped coding pattern (Figure 1a), encoded by “0” and “2” coding particles, is multiplied with a gradient coding sequence “0 1 2 3 0 1 2 3…” (Figure 1b), resulting in the modulus of the mixed coding pattern illustrated in Figure 1c. Note that the four different bright levels (from dark to bright) of the blue color in the coding pattern represent the coding digits (i.e., reflection phases) “0” (0°), “1” (90°), “2” (180°), and “3” (270°), respectively. The analytically calculated scattering patterns clearly show that the original scattering pattern with five beams around the normal axis (Figure 1d) is deflected away from the normal axis without observable distortion (Figure 1f), after conducted by a convolution operation with the single‐beam scattering pattern (Figure 1e).

To better understand the process of scattering‐pattern shift, we give the corresponding frequency spectra, in which the coding patterns are considered as signals in time domain and the scattering patterns are assumed as their corresponding frequency spectra. From Figure 1g, we observe that the original spectrum is located around the zero frequency, which is in analogy to the scattering pattern in Figure 1d. The ideal Dirac function $\delta (\omega -\omega_{0})$ in Figure 1h mimics the single‐beam scattering in Figure 1e and is used to shift the original spectrum to a higher frequency, resulting in the undistorted spectrum at the central frequency ω_0_, as shown in Figure 1i. We remark that an approximation is made in the Dirac function in Figure 1h. Due to the finite size of the coding metasurface, the scattering beam produced by the gradient coding sequence must have a certain width, and hence its corresponding spectrum cannot be treated as a rigorous Dirac function. It will become an ideal Dirac function when the length of the gradient coding sequence approaches to infinity, corresponding to a single‐beam scattering with zero beam width and infinite directivity. However, we will demonstrate in the following examples that such an approximation does not affect the performance of scattering‐pattern shift in real applications.

**Figure 2:**
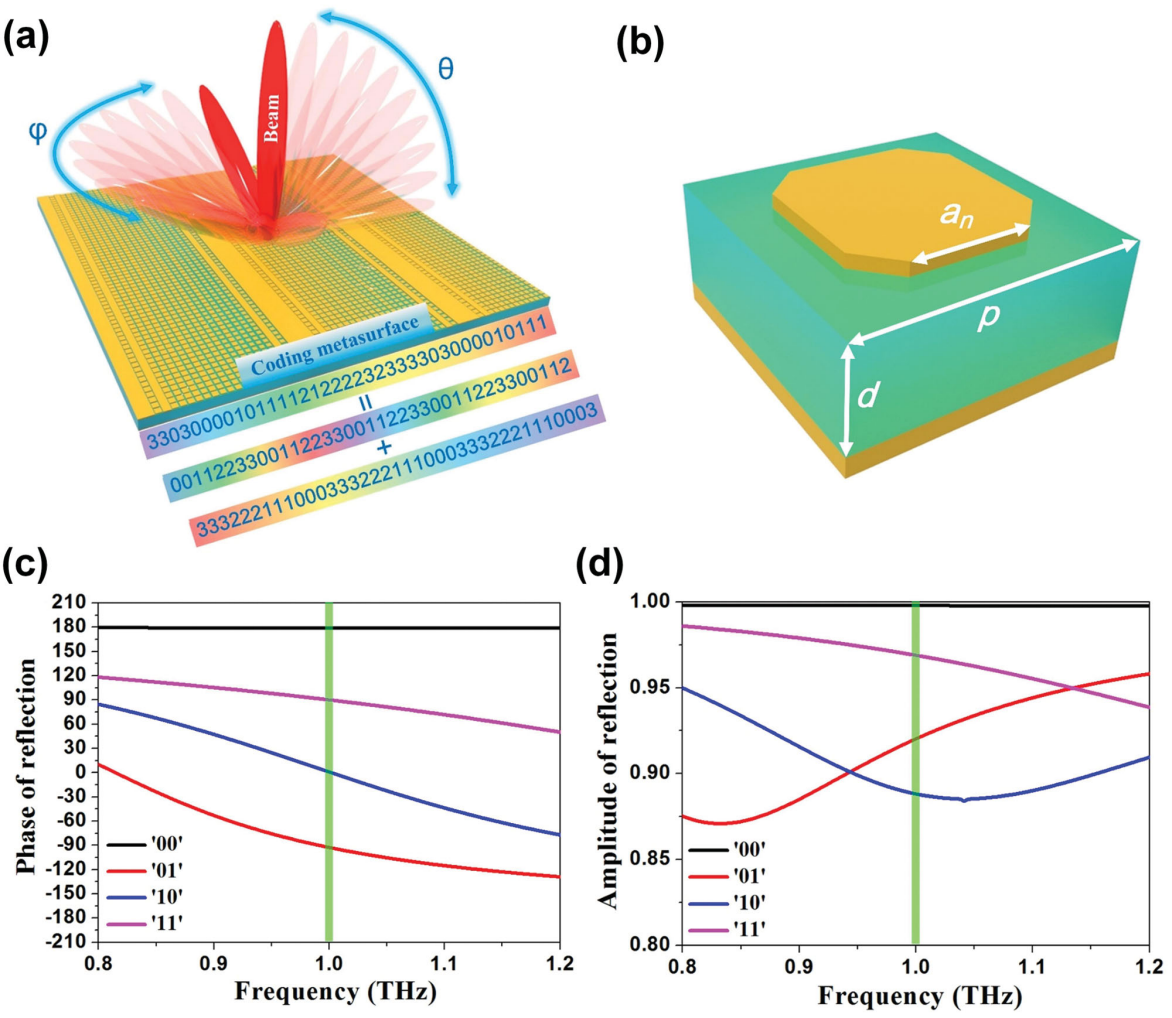
Illustration of the single‐beam scattering to arbitrary directions and the unit cell design. a) Demonstration to the principle of scattering‐pattern shift to produce single‐beam scattering with an arbitrary angle (*θ*, *ϕ*) in the upper‐half space by calculating the modulus of two different gradient coding sequences. b) The structure design of the coding particle. c,d) Reflection phases and amplitudes of the four coding particles “00,” “01,” “10,” and “11,” respectively.

**Figure** **2**a schematically illustrates one of the important applications of this idea to generate a single‐beam scattering pointing in arbitrarily predesigned direction in the upper‐half space, i.e., *θ* from 0° to 90° and *ϕ* from −180° to 180°, as indicated by the red beam. Here, an example is given to demonstrate how the arbitrary direction is realized from two different coding sequences S_1_ (0 0 1 1 2 2 3 3 0 0 1 1 2 2 3 3…) and S_2_ (3 3 3 2 2 2 1 1 1 0 0 0 3 3 3 2 2 2 1 1 1 0 0 0…), each of which generates a single‐beam scattering pointing in a certain direction. For simplicity, the 2‐bit digits “0,” “1,” “2,” and “3” stand for “00,” “01,” “10,” and “11,” respectively. According to the principle of scattering‐pattern shift, the required coding sequence can be calculated by the modulus of them as S_3_ (3 3 0 3 0 0 0 0 1 0 1 1 1 1 2 1 2 2 2 2 3 2 3 3…), which produces a single‐beam pointing in a new direction. Because the added sequence S_2_ is longer in periodicity and with decreasing order, the anomalous scattering angle enabled by the combined sequence S_3_ should be smaller than that from S_1_, as will be discussed in details in the following section.

## 3 Performance Characterization

To validate the principle of the scattering‐pattern shift with realistic materials and structures, we design an octagon‐shaped coding particle at the terahertz frequency, as shown in Figure 2b. It consists of a metallic sheet and metallic octagon‐shaped pattern, separated by a polyimide spacer with thickness *d* = 25 μm and period length *p* = 70 μm. The octagon‐shaped pattern is characterized by side length *a*_n_, with the subscript representing the value of coding digits. For coding particle “00,” the longer side length of the octagon coincides with the edge of the substrate (*a*_0_ = 44 μm). Coding particles “01,” “10,” and “11” are obtained by scaling down the coding particle “00” with ratios 0.844, 0.725, and 0.45 (i.e., *a*_1_ = 37.1 μm, *a*_2_ = 31.9 μm, *a*_3_ = 19.8 μm), respectively. The polyimide is modeled as a dielectric with permittivity *ε* = 3.0 + i0.09. The commercial software, CST Microwave Studio, is used to simulate the reflection phases and amplitudes of the four coding particles, which are shown in Figure 2c,d, respectively. As required by the 2‐bit coding metasurface, the four coding particles “00,” “01,” “10,” and “11” are designed to reflect the normal incident wave with phases of 180°, 90°, 0°, and ‐90° at 1 THz, respectively (see Figure 2c). From Figure 2d, we clearly observe that all amplitudes are larger than 0.86 from 0.8 to 1.2 THz, which ensures the high conversion efficiency of the encoded metasurface.

**Figure 3:**
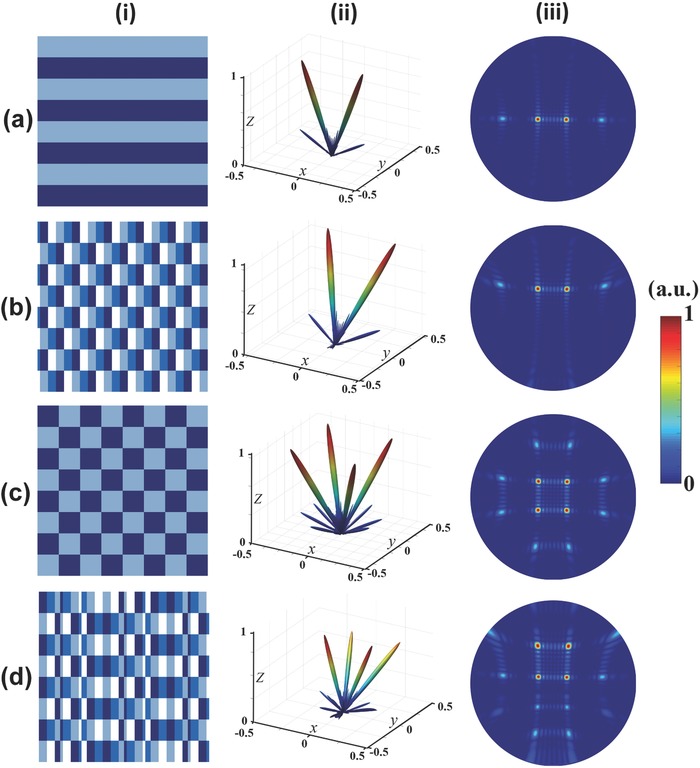
Coding patterns and their 3D and 2D scattering patterns calculated by FFT to show the functionality of perfect steering of scattering pattern. a) Coding pattern of the coding matrix M_1_. b) Coding pattern of the modulus of coding matrix M_1_ and a gradient coding sequence “0 1 2 3 0 1 2 3…” varying along the horizontal direction. c) Coding pattern of the coding matrix M_2_. d) Coding pattern of the modulus of coding matrix M_2_ and the gradient coding sequence. i) Coding patterns. ii) 3D far‐field scattering patterns. iii) 2D far‐field scattering patterns.

To demonstrate the performance of the proposed idea, we give two examples encoded with different coding sequences, as shown in **Figure** **3**. The first example is encoded by a matrix **M_1_** as

(8) $$M_{1}=\left(\begin{array}{cc} 2 & 2\\ 0 & 0 \end{array}\right)$$

where each digit in the coding matrix represents a super unit cell comprising 8 × 8 identical coding particles.^33^ The repetition of identical unit cells is to mimic the periodic boundary condition, so that the EM coupling among adjacent unit cells with different geometrical parameters is minimized. Note that all metasurfaces are encoded with the same number of coding particles of 64 × 64. The coding patterns of **M_1_** are given in Figure 3a(i), in which the colors from dark blue to light blue represent the digits from “00” to “11.” By first calculating the 2D fast Fourier transform (FFT) of the coding pattern, and then making a coordinate transformation, we obtain the corresponding 3D far‐field scattering pattern expressed by the elevation angle *θ* and azimuthal angle *ϕ*, as illustrated in Figure 3a(ii). Please refer to Supporting Information Note S2 for the procedure of calculating the scattering pattern from the coding pattern. The 3D far‐field scattering pattern shows that the normally incident wave is split into two beams symmetrically distributed in the *x–z* plane with respect to the *z*‐axis. To better visualize the scattering angle, Figure 3a(iii) gives the corresponding 2D scattering pattern in a polar coordinate system (see Figure S2c in the Supporting Information for details). From the 2D scattering pattern, we clearly observe two lighter spots close to the center, indicating the two main lobes, and two darker spots far from the center, representing the grating lobes.^38^, ^39^

If a gradient coding sequence “0 1 2 3 0 1 2 3 …” (varying along the horizontal direction, with each coding digit representing 3 × 3 identical coding particles) is added to coding matrix **M_1_**, their modulus gives the mixed coding pattern shown in Figure 3b(i). Carefully observing the mixed pattern, we find that the pattern not only inherits the “0 2 0 2…” coding sequence in the vertical direction, but also gradually varies with the sequence “0 1 2 3 0 1 2 3…” along the horizontal direction. The corresponding 3D scattering pattern in Figure 3b(ii) indicates that the two beams are deviated from the normal axis with the shape of pattern unperturbed, as theoretically expected from the principle of scattering‐pattern shift. The deviation angle from the *z*‐axis is exactly equal to the anomalous scattering angle generated by the gradient coding sequence (see Supporting Information Note S3)

(9) $$\theta =\sin^{-1}\left(\lambda /\Gamma \right)$$

in which *λ* and *Γ* represent the free‐space wavelength and the periodicity of the gradient coding sequence, respectively. Substituting *Γ* = 840 μm and *λ* = 300 μm into Equation (9), the deviated angle is calculated as 20.9°. We should note that in Equation (7), it is not the angle themselves (*θ* and *ϕ*) but the angular components in Equation (3) that are added or subtracted. Since there is no linear relation between *θ* and sinθ, the scattering pattern should, in theory, experiences slight distortion in some extent in this process, especially for large scattering angles. In practical applications, however, the distortion could be neglected due to the approximate linearity between *θ* and sinθ for smaller *θ* values (≤30°).

The next example is given to evaluate the performance of scattering‐pattern shift in controlling multiple beams. The chessboard coding pattern, as shown in Figure 3c(i), is expressed by matrix **M_2_**

(10) $$M_{2}=\left(\begin{array}{cc} 0 & 2\\ 2 & 0 \end{array}\right)$$

in which each digit is composed of 8 × 8 identical coding particles. In this scenario, since the coding sequence is varying along both *x‐* and *y‐*directions, the normally incident wave will be deflected to four directions in the upper‐half space with equal angles *θ* with respect to the *z*‐axis, as observed from the 3D scattering pattern in Figure 3c(ii) and 2D scattering pattern in Figure 3c(iii). The deflection angle *θ* for the chessboard distribution can be predicted by (see Supporting Information Note S3)

(11) $$\theta =\sin^{-1}\left(\sqrt{2}\lambda /\Gamma \right)$$

Substituting *Γ* = 840 μm and *λ* = 300 μm into Equation (11), the deflected angle *θ* is calculated as 22.3°, which is larger than that of the **M_1_** case. Figure 3d(i) illustrates the coding pattern mixed by the chessboard coding matrix and the gradient coding sequence “0 1 2 3 0 1 2 3…,” while Figure 3d(ii,iii) shows the 3D and 2D scattering patterns, respectively. We obviously observe that the whole pattern is tilted away from the *z*‐axis by 20.9°. Again, little distortion can be found in the 2D scattering pattern in Figure 3d(iii), demonstrating the perfect steering of the scattering pattern.

To numerically validate the proposed principle, all coding metasurfaces presented in Figure 3 have been simulated in CST Microwave Studio using the octagon‐shaped structure, as demonstrated in Figure S3 (Supporting Information). Excellent agreements are observed by comparing the FFT predicted scattering patterns in Figure 3 and numerically simulated scattering patterns in Figure S3 (Supporting Information). We remark that the FFT method is able to calculate the far‐field pattern with sufficient accuracy in a few seconds, much less than the numerical simulations for real structures in CST (usually more than 30 minutes and the time will dramatically increase for models having larger electrical length). Such a fast and accurate method for calculating the scattering patterns of coding metasurfaces has greatly reduced the computational complexity and will speed up the optimization of coding metasurfaces in the design of arbitrary beam patterns^40^ and random diffusions.^41^

When the coding metamaterial was first proposed in ref.,^31^ an example was given to reflect the normally incident wave to anomalous direction with a gradient coding sequence “0 1 2 3 0 1 2 3…”^33^ We know from Equation (9) that a smaller periodicity of coding sequence is required to realize a larger anomalous reflection angle, and vice versa. However, based on the previous coding scheme, the attainable reflection angles are limited to a few numbers of values because the periodicity of the coding sequence is multiplied by an integer. Taking the 2‐bit coding metasurface as example, the attainable reflection angles are calculated as 32.4°, 20.92°, and 15.53° when the integer number *n* equals 2, 3, and 4, respectively. As a result, the anomalous reflection angle cannot be arbitrarily designed. For the case when *n* = 1, the length of gradient coding sequence (280 μm) is smaller than the free‐space wavelength (300 μm), in which condition the normally incident wave will not be reflected to space but is converted to the surface wave propagating on the metasurface.^26^

**Figure 4:**
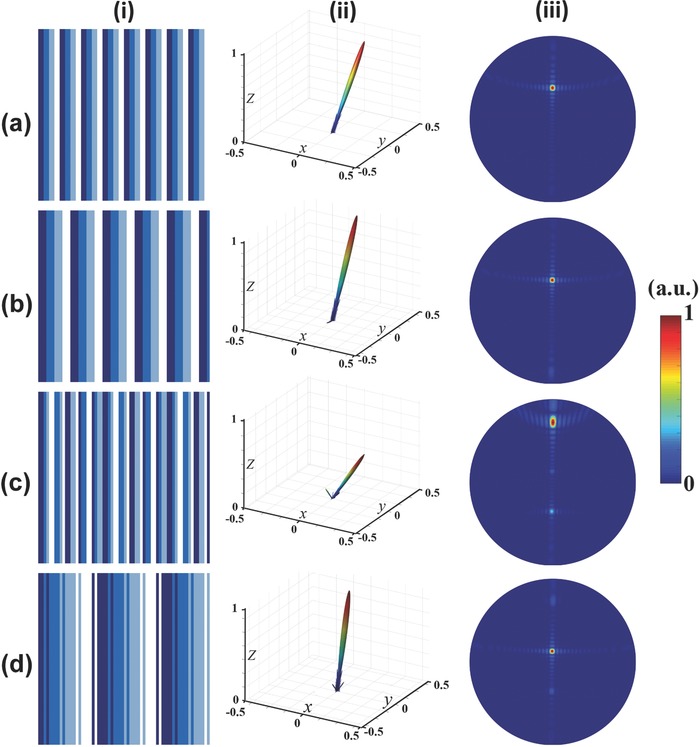
Coding patterns and their 3D and 2D scattering patterns calculated by FFT to show the functionality of a single‐beam scattering to arbitrary direction. a) Coding pattern of the gradient coding sequence “0 0 1 1 2 2 3 3 ….” b) Coding pattern of the gradient coding sequence “0 0 0 1 1 1 2 2 2 3 3 3….” c) Coding pattern of the modulus of gradient coding sequences in panels (a) and (b). d) Coding pattern of the modulus of gradient coding sequences in panel (a) and “3 3 3 2 2 2 1 1 1 0 0 0….” i) Coding patterns. ii) 3D far‐field scattering patterns. iii) 2D far‐field scattering patterns.

Here, we illustrate how the principle of scattering‐pattern shift is used to overcome the above shortcoming and design coding patterns to generate a single beam with arbitrarily scattering angle. **Figure** **4**a(i),b(i) displays the coding patterns of two different coding sequences “0 0 1 1 2 2 3 3…” and “0 0 0 1 1 1 2 2 2 3 3 3…,” respectively. According to Equation (9), there will be anomalous reflections at 32.4° and 20.9° (with respect to the *z*‐axis) in the *x–z* plane, as shown in the 3D/2D scattering patterns in Figure 4a(ii)/(iii),b(ii)/(iii), respectively. Adding such two coding sequences together, we obtain their modulus as shown in Figure 4c(i). It is interesting to note that the mixed coding pattern exhibits a faster variation rate (from digits “0” to “3”) than Figure 4a(i),b(i), and results in a single‐beam scattering with larger angle 63.2°, as displayed by the 3D scattering pattern in Figure 4c(ii). We observe from the 2D scattering pattern in Figure 4c(iii) that the beam spot is close to the edge of the round area, indicating a large reflection angle of the scattering beam. Similarly, when the first coding sequence is subtracted by the second one, it gives the mixed coding pattern shown in Figure 4d(i). As opposed to the coding pattern in Figure 4c(i), the variation rate is slowed down, resulting in the smaller scattering angle of 10.3°, as clearly illustrated in Figure 4d(ii,iii).

**Figure 5:**
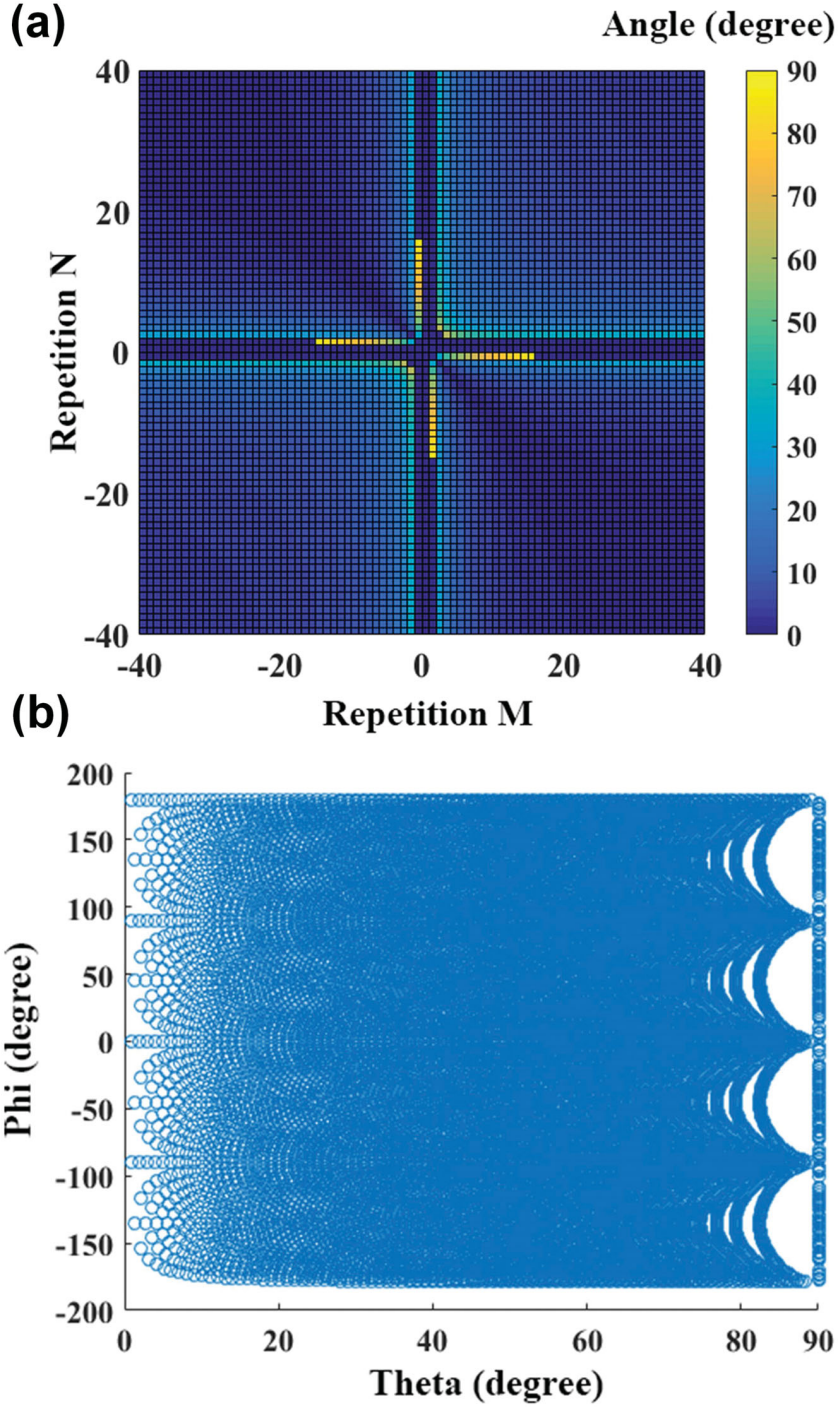
Quantitative analyses of the attainable scattering angles in the elevation and azimuthal planes. a) The calculated scattering angles in the elevation plane obtained from the modulus of two gradient coding sequences varying along the same direction. The numbers M and N in horizontal and vertical axes represent the repetitions of each coding digit in the two gradient coding sequences, and the minus sign indicates the case when the sequence is reversed. b) The scattering angle distribution of the single beam in the upper‐half space (in elevation angle *θ* and azimuthal angle *ϕ*) calculated from the modulus of two gradient coding sequences varying along orthogonal directions (*x‐* and *y‐*direction).

Numerical simulations of structured coding metasurfaces have also been conducted to verify the above theoretical results, as presented in Figure S4 (Supporting Information), in which little discrepancy is observed between the numerical and theoretical results. Supporting Information Figure S5 provides a detailed discussion on the conversion efficiency from the normally incidence to the anomalously scattering beam. We notice that the scattering angles 63.2° and 10.3° are not directly calculated from the addition and subtraction of the scattering angles 32.4° and 20.9°. According to Equation (7), the resulting scattering angles must be calculated in the angular coordinate, as precisely expressed as

(12) $$\theta =\sin^{-1}\left(\sin\theta_{1}\;\;\pm \;\;\sin\theta_{2}\right)$$

in which *θ*_1_ and *θ*_2_ are the anomalous scattering angles corresponding to the two gradient coding sequences that are added or subtracted. To clearly visualize how dense the scattering angle can be realized with this method using the 2‐bit coding metasurface, we calculate the angle *θ* from Equation (12), as plotted in **Figure** **5**a, in which the horizontal and vertical axes represent the repetitions *M* and *N* of each coding digit in the gradient coding sequence “0 1 2 3…,” respectively. For instance, the coding sequence becomes “0 0 1 1 2 2 3 3…” and “0 0 0 1 1 1 2 2 2 3 3 3…” if *M* = 2 and *N* = 3. The minus sign of *M* or *N* indicates the case when the coding sequence is reversed. From Figure 5a, we clearly see that large scattering angles (yellow pixels) are obtained in the vicinity of the center, especially in the second and fourth quadrants where the signs of *M* and *N* are opposite to each other. The scattering angle in the region far from the center is almost below 10° due to the large periodicity of gradient coding sequence. For the current scheme that only two coding sequences are added together, the attainable scattering angles are still not quite densely distributed in the range from 60° to 90°. Fortunately, the density in those directions and the entire upper‐half space can be increased by adding three or multiple coding sequences with different repetition numbers. In the limit of infinitely large number of items being added, the scattering angle can be continuously obtained from 0° to 90°.

The above method allows the scattering beam to scan in the elevation plane. However, in many applications, the beam is required to scan in azimuthal directions, which can be realized by adding a coding sequence grading along the orthogonal direction. Once the anomalous reflection angles generated by two orthogonal gradient coding sequences are given, the new angles *θ* and *ϕ* can be calculated as

(13) $$\{\begin{array}{c} \theta =\sin^{-1}\left(\sqrt{\sin^{2}\theta_{1}\pm \sin^{2}\theta_{2}}\right)\\ \varphi =\tan^{-1}\left(\frac{\sin\theta_{2}}{\sin\theta_{1}}\right) \end{array}$$

in which *θ*_1_ and *θ*_2_ are the elevation angles of the two coding sequences grading along the *x‐* and *y‐*directions. An example is given in Supporting Information Figure S4e to demonstrate the rotation of the beam pattern in the azimuthal direction.

Based on Equations (12) and (13), the attainable scattering angles (*θ,ϕ*) by the modulus of two gradient coding sequences with different repetition numbers (from −90° to 90°, step of 1°) and orthogonal directions (horizontal and vertical) are plotted in Figure 5b. We clearly observe that almost all area in the plot is covered by circles, indicating that the single‐beam scattering is able to scan in the entire upper‐half space with high resolution. Note that the relatively sparse distribution of circles on the left and right edges of the plot, where *θ* is close to 0° and 90°, can be improved by increasing the number of coding sequences being added.

## 4 Experimental and Measured Results

**Figure 6:**
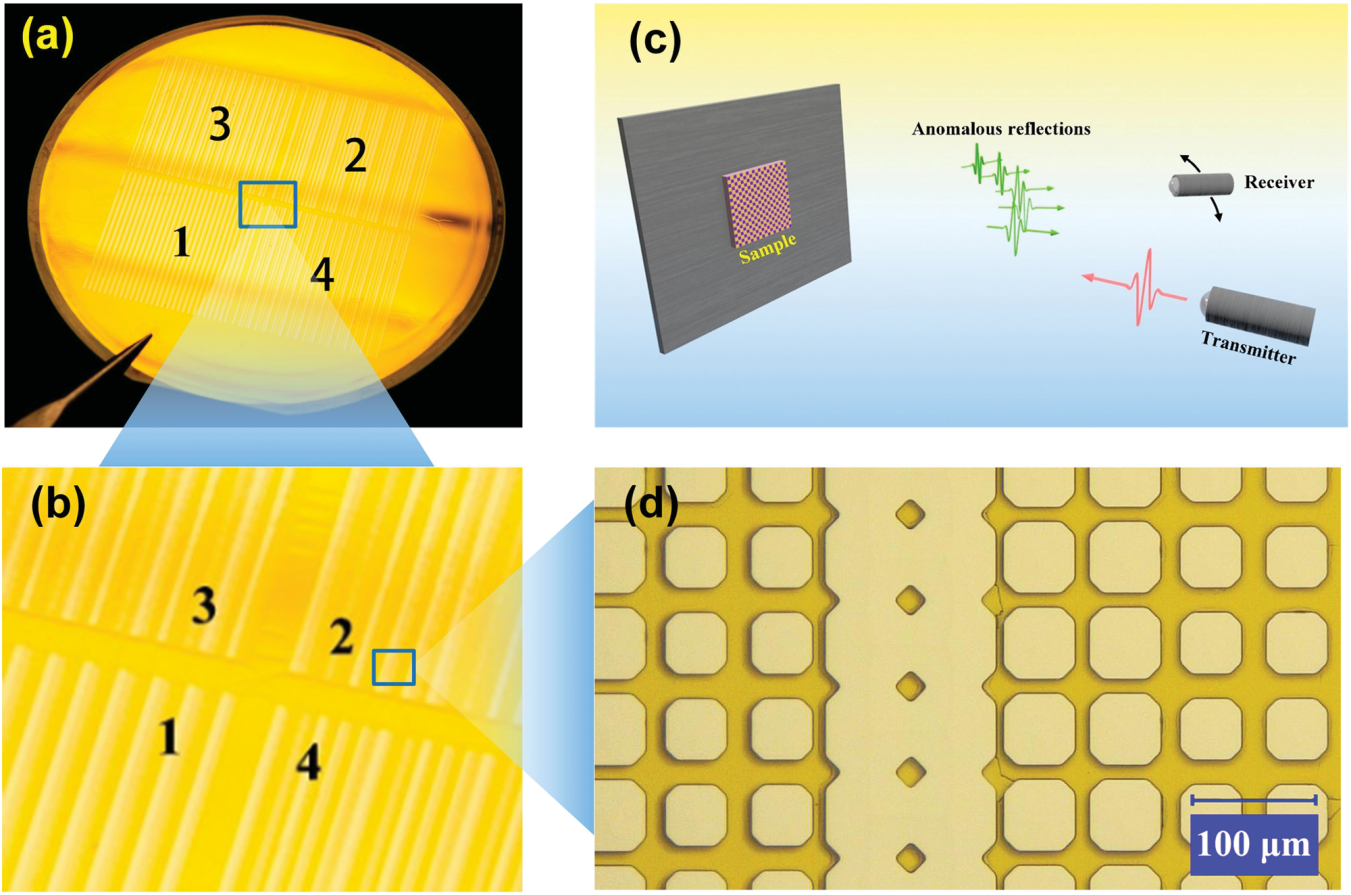
Photographs of the fabricated samples and schematic of the experimental setup. a) Four fabricated samples of terahertz coding metasurfaces labeled with numbers 1, 2, 3, and 4 on the 2 in. silicon wafer, corresponding to coding sequences P_1_, P_2_+P_8_, P_2_+P_4_, and P_2_+P_3_, respectively. b) The enlarged view of the fabricated sample. c) The schematic of the experimental setup for the rotary THz‐TDS. d) The optical microscopy image of the sample.

To experimentally verify the principle of scattering‐pattern shift at the terahertz frequency, four different samples of coding metasurfaces (see **Figure** **6**a,b) were fabricated on a 2 in. silicon wafer using the conventional photolithography. The four different samples, represented by numbers “1,” “2”, “3”, and “4,” were encoded with four different coding sequences P_2_, P_2_+P_8_, P_2_+P_4_, and P_2_+P_3_, respectively, in which P*_n_* represents a gradient coding sequence “0 1 2 3 0 1 2 3…” with the repetition number *n*. For example, P_2_ is “0 0 1 1 2 2 3 3 0 0 1 1 2 2 3 3….” Each sample includes 220 × 220 coding particles, covering an area of 15.4 × 15.4 mm^2^. The microscopy image (VHX‐5000, Keyence Company, Beijing, China) of the sample is shown in Figure 6d. For experimental performance characterization of the fabricated sample, we employed a rotary THz‐TDS system (Zomega Z‐3, East Greenbush, New York, United States) to measure the reflection spectra at different angles, as schematically illustrated in Figure 6c. The radius of the incident terahertz beam (the red arrow) is measured as 5 mm at 1 THz, which is smaller than the fabricated sample. This guarantees that the anomalous reflections (green arrows) from the sample only interact with the sample and are not affected by the surrounding objects (e.g., the gray metallic board).

**Figure 7:**
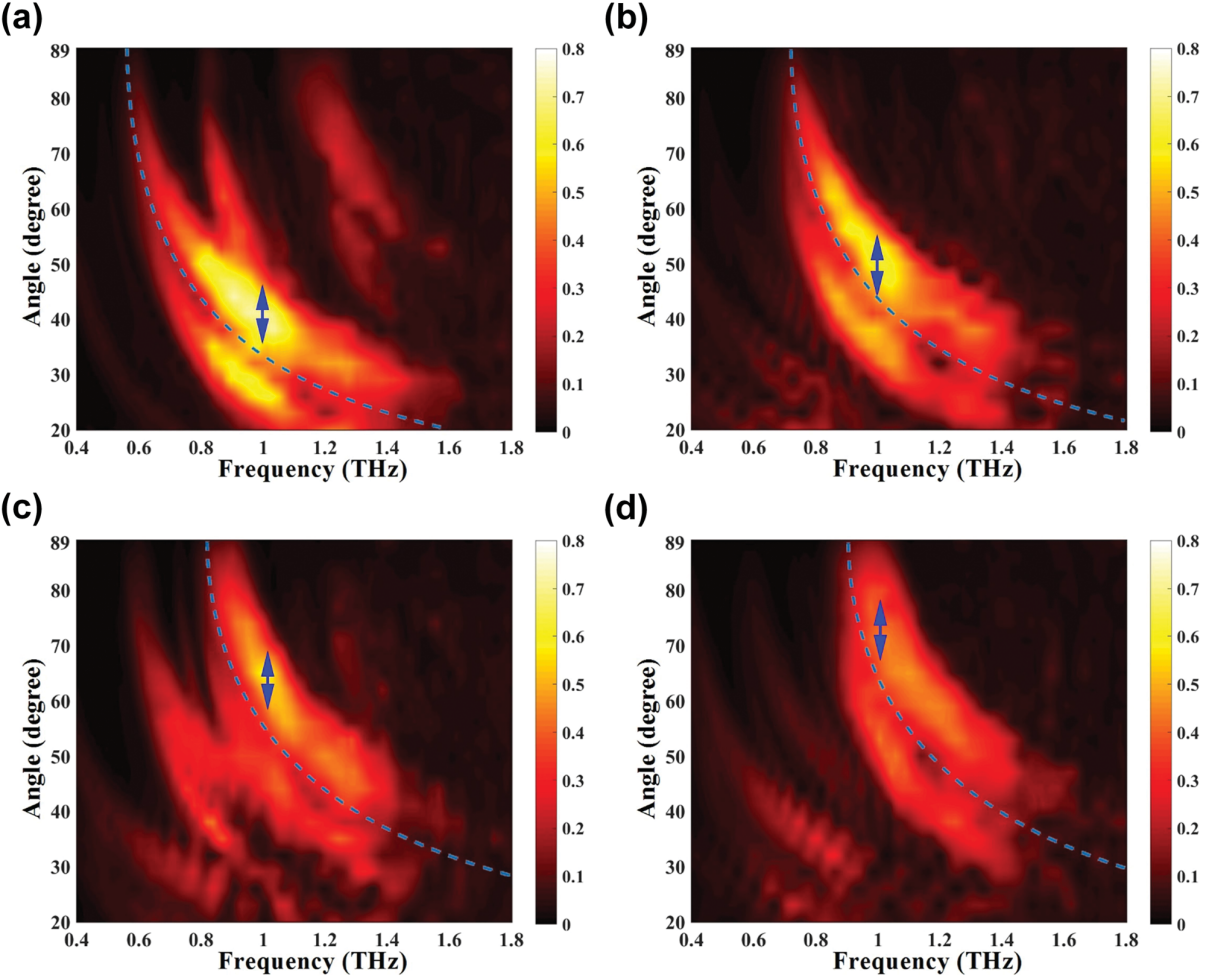
Experimental results of the four fabricated samples encoded with four different coding sequences. The dependence of the normalized reflections on both frequency and receiving angle for coding sequences a) P_2_, b) P_2_+P_8_, c) P_2_+P_4_, and d) P_2_+P_3_. The blue arrow in each plot indicates the angle range of the main‐beam direction at 1 THz. The blue dashed lines indicate the anomalous reflection angles theoretically calculated by Equation (12).

Since the current rotary THz‐TDS system only supports the detection of scattering beams in the horizontal plane, the four fabricated samples are specially designed to generate scattering patterns with a single‐beam pointing in four different directions. **Figure** **7**a–d shows the dependence of the measured reflection amplitudes on both frequency and angle in 2D plots, in which the horizontal and vertical axes represent the frequency and receiving angle, respectively (see Supporting Information Note S5 for data processing). It can be predicted from the above simulations that the faster variation of gradient coding sequence gives the larger scattering angle of the single beam. Since the first case P_2_ has the lowest variation rate among the four cases, the measured amplitude appears in the lowest angle range from 37° to 46° at 1 THz (see the blue arrow). When P_2_ is added with another gradient coding sequence P_8_, it generates a single beam in the directions from 44° to 56°, as observed in Figure 7b, which is larger than the first case. In order to further increase the angle of single‐beam scattering, we add P_4_ and P_3_ to the original coding sequence P_2_, resulting in the scattering peaks appearing at larger ranges of angles from 58° to 68° (Figure 7c) and from 67° to 77° (Figure 7d), respectively.

In order to compare the measured results with the theoretical values, we have plotted in Figure 7 the anomalous reflection angles theoretically calculated by Equation (12) with the blue dashed lines. At the operation frequency of 1 THz, the theoretical reflection angles for the four samples with coding sequences P_2_, P_2_+P_8_, P_2_+P_4_, and P_2_+P_3_ can be read as 32.4°, 42.0°, 53.5°, and 63.2°, respectively. Discussions on the slight discrepancy between the measured and simulated reflection angles can be found in Supporting Information Note S5. We observe from each of the angular‐frequency plots that the scattering peaks tend to shift to smaller angle as the frequency increases, and vice versa. Comparing all the four cases, one may notice that the crescent‐shaped scattering beams are moving toward the upper‐right direction with the increasing variation frequency of the gradient coding sequence. Such two phenomena are in good agreements with the generalized Snell's law,^21^ and can also be verified from Figure S7 (Supporting Information), which gives the reflection amplitudes measured at each receiving angle from 0.4 to 1.6 THz.

The measured efficiencies at the center of scattering peak for the four cases can be obtained from the interpolated data in Figures S7a–d (Supporting Information) as 62%, 61%, 50%, and 40%, respectively. We found that the measured efficiencies decrease with the increasing variation rate of gradient coding sequence. Such a phenomenon can also be observed in the numerical simulations in Figure S5 (Supporting Information), in which the efficiencies are lower for the mixed coding sequences (Figure S5a,b, Supporting Information), especially for larger reflection angles (Figures S5c, Supporting Information). This could be attributed to the following two reasons. First, the periodicity of the mixed coding sequence is longer than those of two constitutive coding sequences. In this case, the periodicities for cases a–d are 8, 12, 24, and 24, respectively, as can be observed from the coding patterns shown in Figure S4(i) (Supporting Information). Therefore, fewer repetitions of the coding sequence will be illuminated by the normal incidence of terahertz waves, leading to the appearances of scattering in other directions. It can be better understood by making an analogy to the Fourier transform in signal processing. A finite‐length sinusoidal signal with lower frequency, i.e., fewer complete sinusoidal waveforms, will have more frequency components in spectrum than the sinusoidal signal of the same length with higher frequency. Second, the momentum matching between the normally incident wave and the obliquely scattered wave becomes worse as the oblique angle increases. Such a momentum mismatch will be much more severe when the anomalous reflection angle approaches 90°, in which circumstance the normally incident beam will be partially converted to surface wave.^26^ Inaccurate value of the thickness and permittivity of the polyimide layer during fabrication will affect the reflection phase of each coding particle, and therefore may also contribute to the discrepancy between the simulation and experimental results.

## 5 Conclusions and Discussion

To summarize, we have proposed a general method to control the scattering of EM waves flexibly and continuously by performing Fourier operations on coding metasurfaces. As a specific operation with convolution, we proposed the principle of scattering‐pattern shift to steer the scattering pattern of a coding metasurface to an arbitrarily predesigned direction. Such a principle to design coding metasurface is inspired by the frequency‐shift property in the conventional Fourier transform theory, and is simply realized by calculating the modulus of the original coding pattern and a gradient coding sequence. This method allows us to rotate the scattering pattern by a certain angle in the 3D space with negligible distortion. One of the most significant contributions of this work is providing a method to realize arbitrary radiation angles based on the 2‐bit coding metasurface which has only four different coding digits. This has overcome the shortcomings of the previous coding scheme,^32^ in which quite a few discrete directions must be obtained due to the use of only one gradient coding sequence. We also showed that the scattering patterns computed from real structures using the numerical software CST are highly consistent with the theoretical results calculated by FFT, which takes far less computing resource and CPU time (by at least three orders of magnitude).

Four coding metasurface samples have been fabricated and measured in the terahertz band to verify the performance of single‐beam scattering to an arbitrary direction. The experimental results show that the normally incident terahertz wave could be deflected to an angle as large as 72° with 40% efficiency, which is significantly better than the previously reported results realized by the conventional reflectarray even at microwave frequency.^42^ Owning to the existence of Fourier transform relation between the coding pattern and far‐field pattern, the coding metasurfaces can be studied from a fully digital perspective, enabling more versatile manipulations to scattering patterns, and therefore have the potential to be applied in many terahertz devices such as metalens,^4^, ^43^ metaholography,^44^, ^45^ and spoof surface plasmon polariton devices.^9^, ^12^, ^46^

We remark that the method proposed in this work is not limited to the terahertz band, but can be easily extended to the microwave and optical frequencies. Further studies will focus on the implementation of programmable anisotropic metasurfaces using active elements, such as varactor diodes^33^ in the microwave freuqnecy or doped silicon^47^ in the terahertz frequency, to generate various beam patterns in real time.

## 6 Experimental Section

*Sample Fabrication*: The fabrication process mainly included the preparation of a polyimide layer and the conventional photolithography. First, a 180 nm thick gold layer was e‐beam‐evaporated on a 2 in. silicon wafer (400 μm thick, n‐type, resistivity *ρ* = 3–6 Ω·cm) to serve as the metallic background. Then, a 25 μm thick polyimide layer was coated on the gold layer and baked on a hot plate at 80, 120, 180, and 250 °C for 5, 5, 5, and 20 min, respectively. Since the liquid polyimide chosen (Yi Dun New Materials Co. Ltd, Suzhou) could only be used to form a maximum thickness of 10 μm at the minimum spin rate of 1150 rpm, the above spin‐coating and curing processes were repeated twice for the final completion of the 25 μm thick polyimide layer. Finally, followed by the standard photolithography, another Ti/Au layer (10/180 nm) was deposited on the patterned photoresist by electron‐beam evaporation. The final metallic pattern was fabricated by a liftoff process in acetone.

*Measurement Systems*: The reflection spectra of the fabricated samples with a broadband (0.3–3.0 THz) 4‐*f* fiber‐based rotary THz‐TDS were measured, as schematically illustrated in Figure 6c. Here, two fiber‐based terahertz photoconductive antennas (TR4100‐RX1, API Advanced Photonix, Inc.), optically excited by a commercial ultrafast erbium fiber laser system (T‐Gauge, API Advanced Photonix, Inc.), were used to generate and receive the vertically polarized terahertz signals in time domain. The transmitter was fixed on the optical stabilization platform, while the receiver was mounted on a rotary stage, which allows the receiver to rotate around the central cylinder with high precision (see Figure S6, Supporting Information). The system had a signal‐to‐noise ratio of about 27 dB at the designed frequency 1 THz (see Supporting Information Note S5). The sample was attached to a metallic board mounted on the central cylinder. Both the transmitter and receiver were kept at the same distance of 23 cm to the sample. Due to the blockage of both antennas at smaller angle, the minimum receiving angle was limited at 20°. In the measurement, the receiver was rotated from 20° to 89° in the horizontal plane with a step of 3° to record the electric fields scattered from the sample. The direct transmission recorded when the transmitter and receiver were placed in a straight line was used as the reference for the four cases.

## Supporting information

As a service to our authors and readers, this journal provides supporting information supplied by the authors. Such materials are peer reviewed and may be re‐organized for online delivery, but are not copy‐edited or typeset. Technical support issues arising from supporting information (other than missing files) should be addressed to the authors.

Supplementary
