# Supplementary material for: Convolution Operations on Coding Metasurface to Reach Flexible and Continuous Controls of Terahertz Beams
Source: Adv Sci (Weinh). 2016 Jul 7;3(10):1600156. doi: 10.1002/advs.201600156 (PMC5096125; doi:10.1002/advs.201600156)
Supplement: Supplementary file 1 — Supplementary [file ADVS-3-0n-s001.pdf]

Copyright WILEY-VCH Verlag GmbH & Co. KGaA, 69469 Weinheim, Germany, 2016.

ADVANCED SCIENCE
Open Access

Supporting Information

for *Adv. Sci.,* DOI: 10.1002/advs.201600156

Convolution Operations on Coding Metasurface to Reach Flexible and Continuous Controls of Terahertz Beams

Shuo Liu, Tie Jun Cui, Lei Zhang, Quan Xu, Qiu Wang, Xiang Wan, Jian Qiang Gu, Wen Xuan Tang, Mei Qing Qi, Jia Guang Han, Wei Li Zhang, Xiao Yang Zhou, and Qiang Cheng*

Supporting information:

Convolution operations on coding metasurface to reach flexible and continuous controls of terahertz beams

Shuo Liu, Tie Jun Cui*+, Lei Zhang, Quan Xu, Qiu Wang, Xiang Wan, Jian Qiang Gu, Wen Xuan Tang, Mei Qing Qi, Jia Guang Han, Wei Li Zhang, Xiao Yang Zhou, and Qiang Cheng*

Shuo Liu*, Prof. Tie Jun Cui*, Lei Zhang, Xiang Wan, Wen Xuan Tang, Mei Qing Qi, Xiao Yang Zhou, Qiang Cheng
State Key Laboratory of Millimeter Waves, Southeast University, Nanjing 210096, China
+Corresponding author: E-mail: tjcui@seu.edu.cn
* These authors contribute equally to this work.

Shuo Liu, Lei Zhang, Xiang Wan, Wen Xuan Tang, Mei Qing Qi
Synergetic Innovation Center of Wireless Communication Technology, Southeast University, Nanjing 210096, China

Prof. Tie Jun Cui, Qiang Cheng, Wei Li Zhang
Cooperative Innovation Centre of Terahertz Science, No.4, Section 2, North Jianshe Road, Chengdu 610054, China

Quang Xu, Qiu Wang, Jian Qiang Gu, Jia Guang Han, Wei Li Zhang
Center for Terahertz Waves and College of Precision Instrument and Optoelectronics Engineering, Tianjin University, Tianjin 300072, China

Xiao Yang Zhou
Jiangsu Xuantu Technology Co., Ltd., 12 Mozhou East Road, Nanjing 211111, China

Keywords: coding metasurface, Fourier transform, convolution theorem, scattering-pattern shift, terahertz

The PDF file includes:

- Note S1. Derivation for the principle of scattering-pattern shift.
- Note S2. Procedure of calculating the scattering pattern from coding pattern.
- Note S3. Deviation of the anomalous scattering angle.
- Note S4. Numerical simulation results from CST.
- Note S5. Experimental results.
- Figure S1. A coding metasurface located on the x-y plane in the Cartesian coordinate system.
- Figure S2. The process of obtaining the far-field scattering pattern from the coding pattern using the FFT algorithm.
- Figure S3. Geometries of coding patterns and the corresponding 3D and 2D scattering patterns that are numerically simulated by CST to verify the theoretical results in Figure 3.
- Figure S4. Geometries of coding patterns and the corresponding 3D and 2D scattering patterns that are numerically simulated by CST to verify the theoretical results in Figure 4.
- Figure S5. The scattering patterns extracted from the x-z cutting-plane in the 3D scattering patterns in Figure S4.
- Figure S6. Photograph of the terahertz experimental configuration.
- Figure S7. The reflection spectra measured at receiving angles from 20° to 86° of the four fabricated samples encoded with four different coding sequences.

Note S1. Derivation for the principle of scattering-pattern shift

We take 1-bit coding metasurface as example to elaborate the process of calculating the scattered electric field pattern $\boldsymbol{E}(\theta, \varphi)$ in the far-field region directly from the near electric field distribution $E(x, y)$ on the coding pattern. As schematically illustrated in Supplementary Figure S1, the yellow and blue patches represent the coding particles '0' and '1', respectively. For simplicity, we consider a case when the tangential electric field on the metasurface has only x-component $\boldsymbol{E}(x, y) = \hat{x}E(x, y)$, which can be easily extended to the general case $\boldsymbol{E}(x, y) = \hat{x}E_x(x, y) + \hat{y}E_y(x, y)$. The reflection amplitude is assumed to be unity due to the perfect reflection of the coding particle, and thus the electric field on the metasurface can be expressed in the Cartesian coordinate system as

$$E_{m,n}(x', y') = e^{i\varphi(m,n)} \tag{S1}$$

in which $\varphi(m, n)$ represents the reflection phase of coding particle (m,n), which is either 0 or π; and x', y' are limited to the size of a coding particle ($-\frac{p}{2} < x', y' < \frac{p}{2}$).

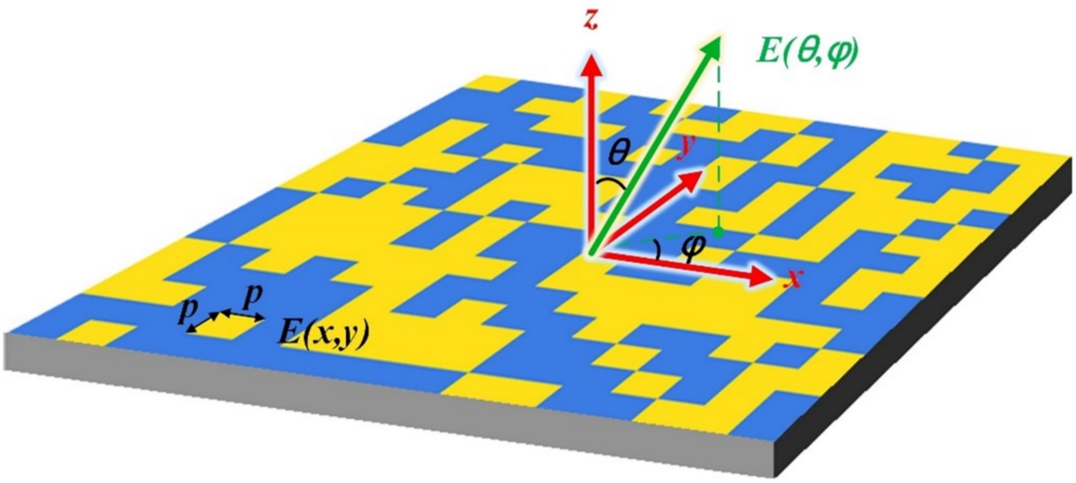

Figure S1. A coding metasurface located on the x-y plane in the Cartesian coordinate system.

For an encoded metasurface board with N×N coding particles, the electric field in upper-half

space can be written by double integrals using the angular spectrum of plane waves. Then, the electric field in the far-field region can be expressed as follow in the spherical coordinate system by making an asymptotic evaluation of the integrals using the stationary phase approximation:

$$\boldsymbol{Es}(\theta,\varphi) = jk(\hat{\theta}\cos\varphi - \hat{\varphi}\sin\varphi\cos\theta)\int_{-\frac{Np}{2}}^{\frac{Np}{2}}\int_{-\frac{Np}{2}}^{\frac{Np}{2}} E(x,y)e^{jk_0(ux+vy)}dxdy$$

$$= jk(\hat{\theta}\cos\varphi - \hat{\varphi}\sin\varphi\cos\theta)P(u,v) \quad \text{(S2)}$$

in which $P(u,v)$ is the Fourier transform of $E(x,y)$, and (u,v) are the angular coordinates: $u = \sin\theta\cos\varphi$, $v = \sin\theta\sin\varphi$. To evaluate the integral in Equation (S2) by each coding particle, the relation between coordinates (x,y) and (x',y') is defined as

$$x = x' + mp - \frac{(N-1)p}{2} \quad \text{(S3)}$$

$$y = y' + np - \frac{(N-1)p}{2} \quad \text{(S4)}$$

Substituting Equation (S3) and (S4) into $P(u,v)$, the spectral function is rewritten as

$$P(u,v) = K \cdot \sum_{m=0}^{N-1}\sum_{n=0}^{N-1}\left[e^{jk_0(ump+vnp)}\right] \cdot \int_{-\frac{p}{2}}^{\frac{p}{2}}\int_{-\frac{p}{2}}^{\frac{p}{2}} E_{m,n}(x',y')e^{jk_0(ux'+vy')}dx'dy' \quad \text{(S5)}$$

where

$$K = e^{-j\frac{k_0(N-1)p(u+v)}{2}} \quad \text{(S6)}$$

For both coding particles, the amplitude and phase of the reflected electric field are assumed to be uniformly distributed in the square area. Then we may rewrite Equation (S5) by integrating on the coding particles

$$P(u,v) = K \cdot p^2 sinc\left(\frac{k_0 up}{2}\right) sinc\left(\frac{k_0 vp}{2}\right) \sum_{m=0}^{N-1} \sum_{n=0}^{N-1} \left[e^{i\varphi(m,n)} e^{jk_0(ump+vnp)} \right] \quad \text{(S7)}$$

Now the spectral function is simplified to a double summation on the coding metasurface with $N \times N$ coding particles. Interestingly, the double summation can be easily calculated with the 2D inverse discrete Fourier transform

$$f(s,t) = FFT2[F(m,n)] = \frac{1}{N^2} \sum_{m=0}^{N-1} \sum_{n=0}^{N-1} F(m,n) e^{j\frac{2ms\pi+2nt\pi}{N}} \quad \text{(S8)}$$

where a finite-sized matrix $\mathrm{f}(s,t)$ composed of equally spaced samples is converted into a matrix of coefficients with finite combination of complex sinusoids, ordered by their frequencies. Applying the above 2D FFT algorithm to the spectral function in Equation (S2), it can be obtained in a discrete number of angular coordinates (u,v)

$$u = \frac{2\pi}{Npk_0} s \qquad s = 0,1,2, \dots N-1 \quad \text{(S9)}$$

$$v = \frac{2\pi}{Npk_0} t \qquad t = 0,1,2, \dots N-1 \quad \text{(S10)}$$

Now, the scattering pattern in the far field is obtained in terms of (θ,φ) component by calculating the spectral function $P(u,v)$ using the 2D FFT

$$E_\theta = jk \frac{e^{-jkr}}{2\pi r} P(u,v) \cos\varphi \quad \text{(S11)}$$

$$E_\varphi = -jk \frac{e^{-jkr}}{2\pi r} P(u,v) \cos\theta \sin\varphi \quad \text{(S12)}$$

Note S2. Procedure of calculating the scattering pattern from coding pattern

We should note that, when applying 2D FFT algorithm, the scattering pattern can be evaluated in a number of $u \times v$ points, which are equal to the number of coding particles on the coding metasurface

($N \times N$). However, one may calculate the far-field scattering patterns in a larger number of $u \times v$ points by extending the size of the coding metasurface and setting the amplitude of all coding particles outside the designed region as zero.

By making a shift in the variable (s, t) and (m, n) in Equation (S9) and (S10), (u, v) have the upper and lower bounds of $-\frac{\lambda}{2\mathrm{p}} < \mathrm{u}, \mathrm{v} < \frac{\lambda}{2\mathrm{p}}$. We note that the whole visible range of (θ, φ) should be mapped from the inner area of the circle $u^2 + v^2 \leq 1$ (marked by the pink circular area in Supplementary Figure S2b) in the (u, v) region. Therefore, the periodic length p of coding particle will play a significant role in the coordinate transformation from (u, v) to (θ, φ). When the length p of the coding particle is equal to or smaller than $\lambda/2$, the data inside the circle $\mathrm{u}^2 + \mathrm{v}^2 \leq 1$ will be mapped to the (θ, φ) region to produce the scattering patterns containing the entire range of visible angles. The data outside the circle, in this case, will not be transformed to the visible angles in spherical coordinate system. On the contrary, if $\mathrm{p} > \lambda/2$, then not all of the visible angles will be obtained since the side length of the square region (u, v) is smaller than the diameter of the circle. For the coding particle in this work, $p = 7\lambda/30$ is smaller than the threshold value $\lambda/2$, guaranteeing the ability of the scattering to cover the entire upper-half space.

Note S3. Deviation of the anomalous scattering angle

Now we give the angle derivation of the single-beam scattering by the gradient coding sequence from the perspective of Fourier transform. In analogy to the Fourier transform between the electric field distribution on the coding metasurface and far-field scattering, we can make the following variable substitutions to give the frequency-shift function in Equation (7)

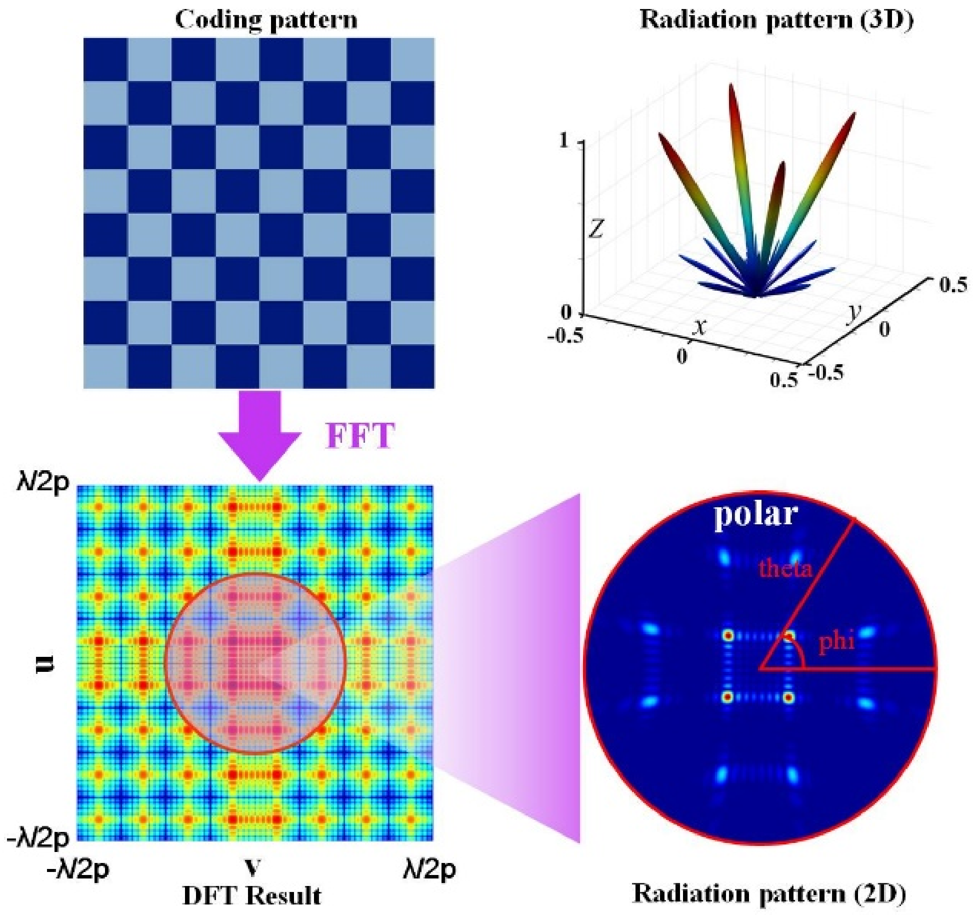

Figure S2. The process of obtaining the far-field scattering pattern from the coding pattern using the FFT algorithm. a) The coding pattern. b) The FFT result of the coding pattern. c) The 2D scattering pattern plotted in the polar coordinate system, in which the elevation and azimuthal directions represent the angles θ and φ, respectively. d) The 3D far-field scattering pattern plotted in the spherical coordinate system.

$$t \to x_\lambda = \frac{x}{\lambda} \tag{S13}$$

$$\omega \to sin\,\theta \tag{S14}$$

Since ω is equal to 1/t, then we have $\sin\theta = \lambda/x$. For a metasurface encoded with an infinite-long gradient coding sequence with periodicity Γ, the corresponding Dirac function, representing the frequency of the coding sequence (or phase) grading along the metasurface, will appear at λ/Γ.

Therefore, the electric field in the far region will strictly pointing to the direction $\theta = \sin^{-1}(\lambda/\Gamma)$, which is the anomalous scattering angle defined in Equation (9). However, for real applications where the periodicity of gradient coding sequence is finite, the ideal Dirac function will be degenerated as a sharp peak function with finite width and amplitude, producing the single-beam scattering with a certain lobe width.

As for where the item $\sqrt{2}$ comes from in Equation (11), we note that the chessboard distribution shown in Figure 3c(i) can be seen as the modulus of two orthogonal coding sequences '0 1 0 1…'. Since such two coding sequences are identical and orthogonal to each other, the new scattering angle (θ, φ) of the modulus pattern (i.e., the chess-board distribution) can be calculated by substituting $\theta_1 = \theta_2$ into Equation (13):

$$\begin{cases} \theta = \sqrt{2}\theta_1 \\ \varphi = 45° \end{cases} \tag{S15}$$

It is obvious that the elevation angle in this case is $\sqrt{2}$ times larger than that with single gradient coding sequence.

Note S4. Numerical simulation results from CST

To confirm the performance of the proposed principle using full-wave numerical simulations, the commercial software CST Microwave Studio was employed to design the structure of the coding particle and simulate the model of encoded metasurface. Frequency domain solver with a unit-cell boundary condition was used to calculate the reflection amplitude and phase of each coding particle, which are shown in Figures 2c,d, respectively. Note that both amplitudes and phases of reflection are extracted on the top surface of the structure.

Open boundary condition and plane-wave excitation was set in the simulation of the encoded metasurface including 64×64 coding particles. The geometrical models of the coding patterns, 3D and 2D scattering patterns are provided in Figures S3 and S4, respectively, which correspond to the theoretical results given in Figures 3 and 4. Due to the fact that the intensities of the 3D/2D scattering patterns in Figures S3 and S4 may vary from case to case, they have been normalized to their own maximum values so that all the scattering patterns are with appropriate color and size for readers to observe.

Figure S4e(i) displays the mixed coding pattern generated by the modulus of two coding sequences ‘0 0 1 1 2 2 3 3’ (grading along the *x*-axis) and ‘0 0 0 1 1 1 2 2 2 3 3 3’ (grading along the *y*-axis). The gradient coding sequence is observed to vary along an oblique direction (33.7°) with respect to the *y*-axis, which can be calculated by Equation (13). The resulting 3D and 2D scattering patterns are presented in Figure S4e(ii) and Figure S4e(iii), respectively, in which the single scattering beam is observed to appear in the direction of θ=40° and φ=123.7°. We should note that the addition of two gradient coding sequences with orthogonal gradient directions is equivalent to rotating the matrix of a gradient coding sequence by a certain angle.

In the main context, we have demonstrated the excellent performance of scattering-pattern shift in steering the scattering pattern to arbitrary direction. Now, we will evaluate the efficiency of the single-beam scattering quantitatively, which is widely considered by engineers in real applications. To compare the efficiencies of the four cases in Supplementary Figures S4a-d, we firstly extract the 2D scattering patterns on the *x*-*z* cutting-plane and then normalize them to the maximum specular reflection by a bare PEC board, which has the same dimension to the above

cases and is titled by $\theta/2$ (θ is the reflection angle of the single beam in each case) with respect to the z-axis. Now, the maximum amplitude of each pattern in Supplementary Figure S5 represents the conversion efficiency from the normally incident plane wave to the anomalously reflected beam. The efficiencies for the four cases in Supplementary Figure S4a-d can be read from Supplementary Figure S5 as 86.6%, 80.3%, 57.5%, and 75.2%, respectively. We should note that the single-beam scattering always has a certain beam width because of the finite size of the coding metasurface. Therefore, the efficiencies shown above only consider the amplitude at the maximum radiation direction. However, one may also evaluate the conversion efficiency by integrating the energy in a certain solid angle of the beam. In this way, the conversion efficiency may vary depending on the limit of the integration angle.

Note S5. Experimental results

Using the terahertz measurement system shown in Supplementary Figure S6, we obtain the reflection spectra for each receiving angle, as illustrated in Supplementary Figure S7, by the following procedure. First, a fast Fourier transform (FFT) was conducted to the original discretized signal in the time domain, resulting in the original spectra in the frequency domain. Then, all spectra were normalized to the reference measured under direct transmission, after which a smooth treatment was carried out to eliminate the background noise, which is at about -58 dB for our measurement system. Since the amplitude of the THz signal generated by the photoconductive antenna decreases with the increasing of frequency, the signal-to-noise (SNR) ratio ranges from 57 dB to 10 dB in the frequency band of interest (from 0.4 to 1.4 THz) and reaches 27 dB at the designed frequency 1 THz. The angular-frequency spectra given in Figure 7 were produced by

making a data interpolation to the spectra in Supplementary Figure S7.

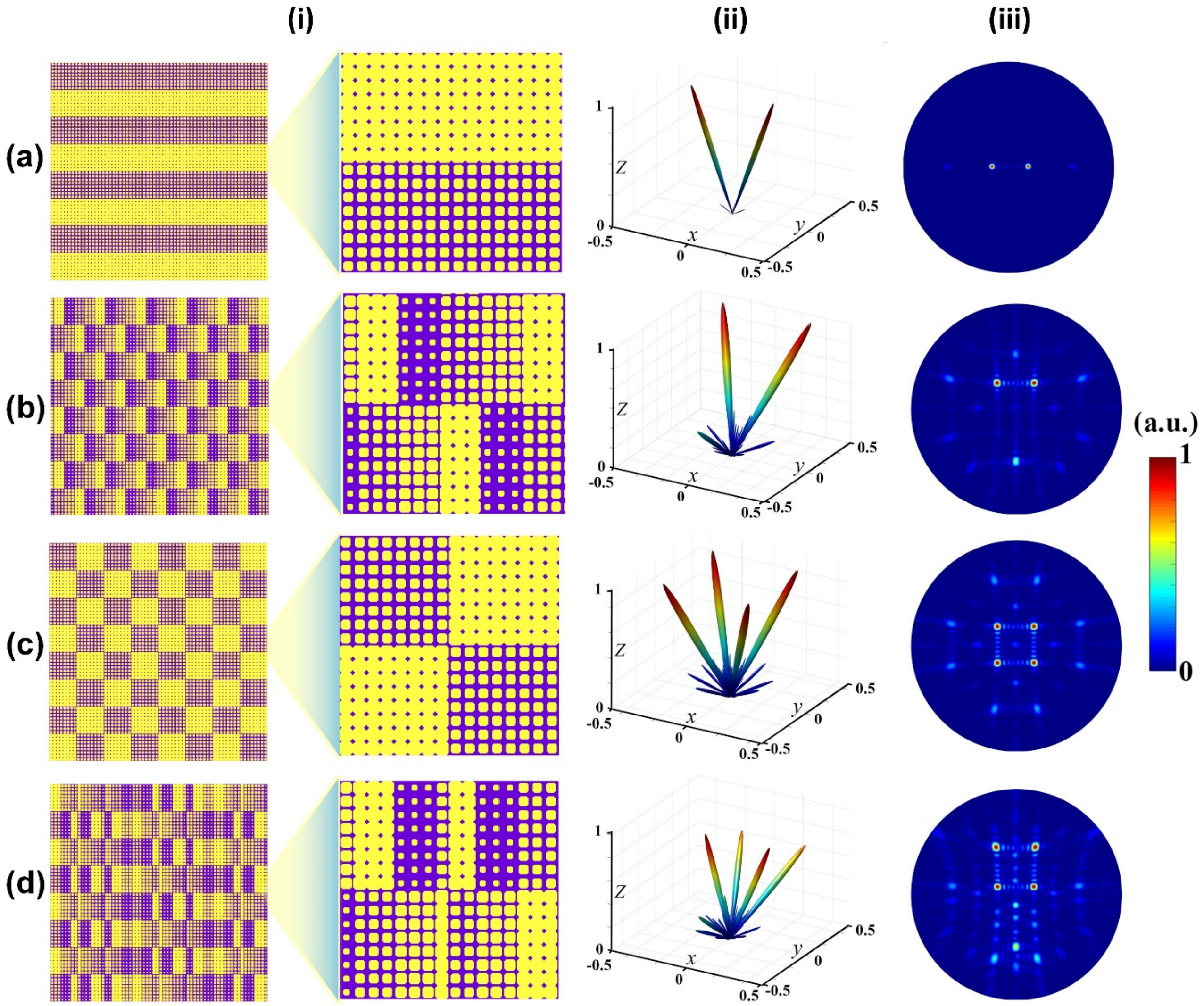

Figure S3. Geometries of coding patterns and the corresponding 3D and 2D scattering patterns that are numerically simulated by CST to verify the theoretical results in Figure 3. a) The coding pattern of the coding matrix M_1. b) The coding pattern of the modulus of coding matrix M_1 and a gradient coding sequence '0 1 2 3 0 1 2 3…' varying along the horizontal direction. c) The coding pattern of the coding matrix M_2. d) The coding pattern of the modulus of coding matrix M_2 and a gradient coding sequence '0 1 2 3 0 1 2 3…' varying along the horizontal direction. (i) Coding patterns. (ii) 3D far-field scattering patterns. (iii) 2D far-field scattering patterns.

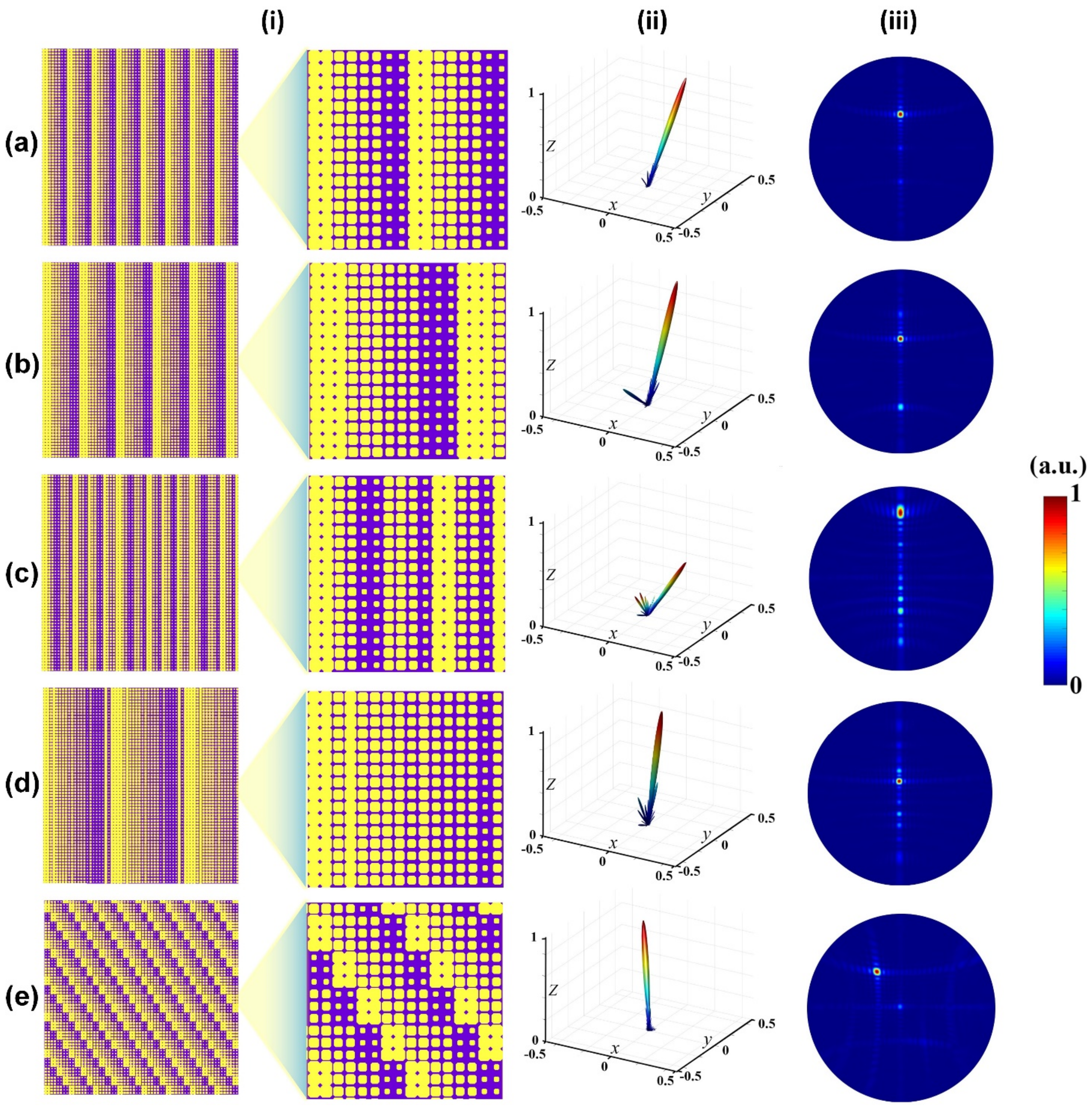

Figure S4. Geometries of coding patterns and the corresponding 3D and 2D scattering patterns that are numerically simulated by CST to verify the theoretical results in Figure 4. a) Coding pattern of the gradient coding sequence ‘0 0 1 1 2 2 3 3 …’ b) Coding pattern of the gradient coding sequence ‘0 0 0 1 1 1 2 2 2 3 3 3…’ c) Coding pattern of the modulus of gradient coding sequences in (a) and (B). d) Coding pattern of the modulus of gradient coding sequences ‘0 0 1 1 2 2 3 3 …’ and ‘3 3 3 2 2 2 1 1 1 0 0 0…’ e) Coding pattern of the modulus of gradient coding sequences ‘0 0 1 1 2 2 3 3 …’ (along the *x*-axis) and ‘0 0 0 1 1 1 2 2 2 3 3 3…’ (along the *y*-axis). (i) Coding patterns. (ii) 3D

far-field scattering patterns. (iii) 2D far-field scattering patterns.

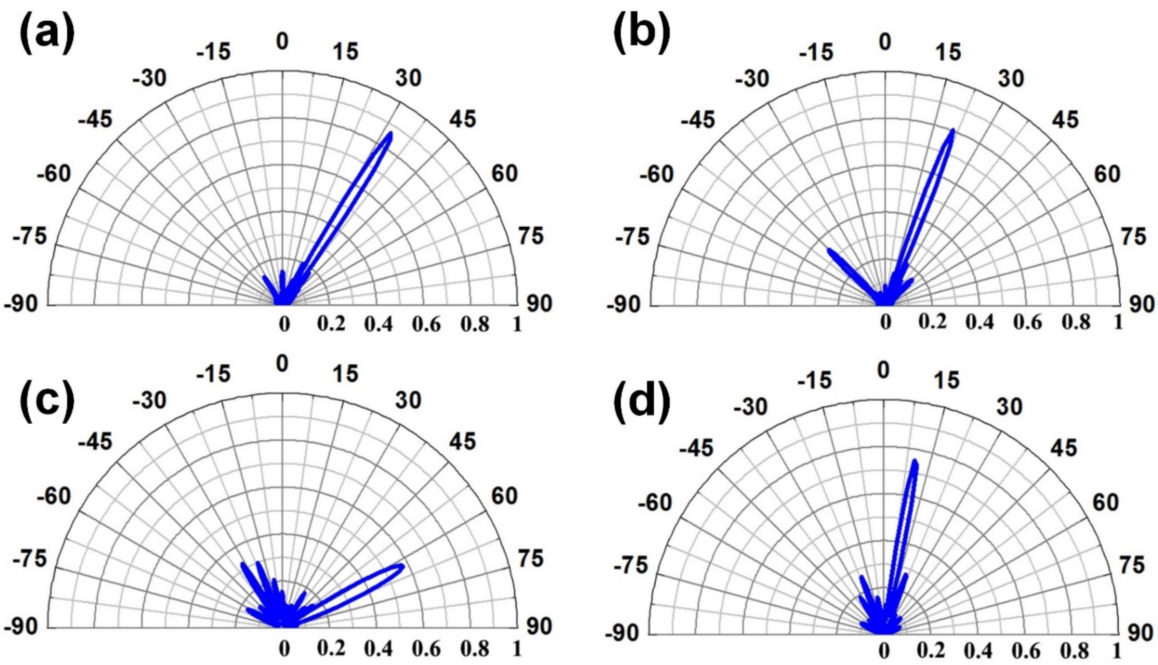

Figure S5. The scattering patterns extracted from the *x-z* cutting-plane in the 3D scattering patterns in Figure S4. a-d) Corresponding to Figures S4(a-d), respectively.

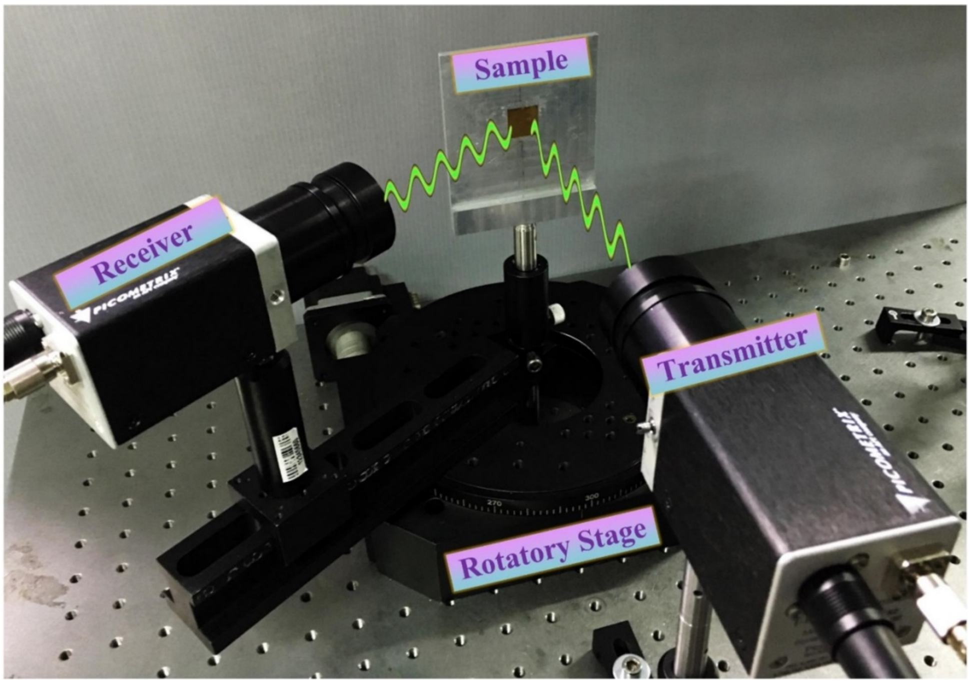

Figure S6. Photograph of the terahertz experimental configuration.

Observing each case in Supplementary Figure S7, we find that the scattering peaks gradually shift to lower frequencies as the receiving angle increases. Comparing all the four cases from Figures S7a to S7d, all scattering peaks tend to move towards higher frequencies due to the increasing of the variation rate of the gradient coding sequence. The center of the scattering beam can be read from Figure S7a-d as 41.5°, 50°, 63°, and 72°, which are about 9° larger than the theoretically calculated values 32.4°, 42°, 53.5°, and 63.2°, respectively. We need to note that the scattering angle at 1.2 THz is close to the theoretical result, which implies that the larger measured angle of scattering beam is equivalent to the frequency-shift of spectrum.

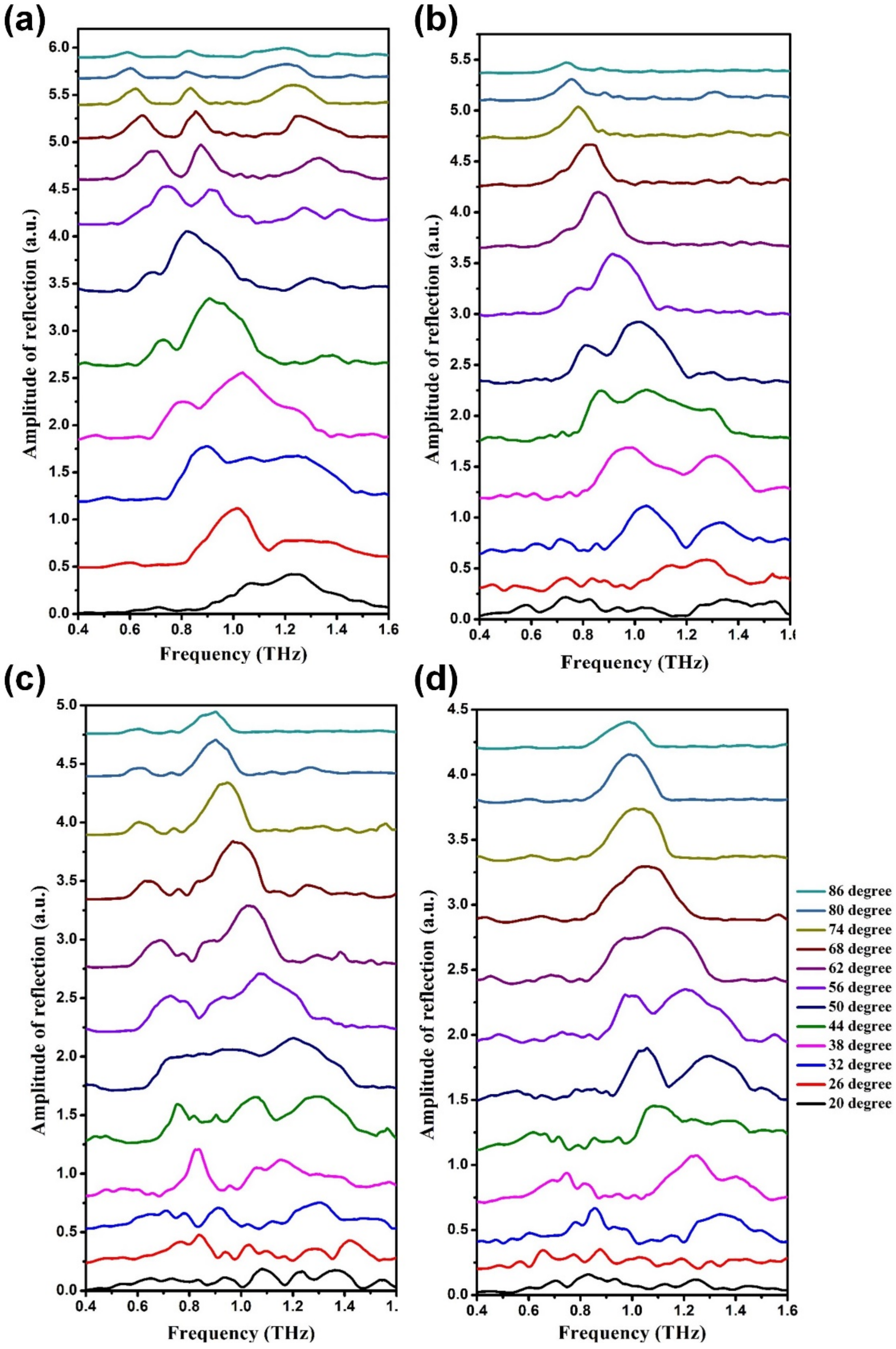

Figure S7. The reflection spectra measured at receiving angles from 20° to 86° of the four fabricated samples encoded with four different coding sequences. a-d) The reflection spectra corresponding to the coding sequences P_1, P_2+P_8, P_2+P_4, and P_2+P_3, respectively.
